# The effect of pacifier sucking on orofacial structures: a systematic literature review

**DOI:** 10.1186/s40510-018-0206-4

**Published:** 2018-03-13

**Authors:** Karin Michèle Schmid, Remo Kugler, Prasad Nalabothu, Carles Bosch, Carlalberta Verna

**Affiliations:** Department of Orthodontics and Pediatric Dentistry, University Center for Dental Medicine Basel, Hebelstrasse 3, 4056 Basel, Switzerland

**Keywords:** Malocclusion, Pacifier, Non-nutritive sucking habits, Orofacial structures, Overjet, Open bite, Posterior crossbite, Systematic review

## Abstract

**Background:**

Non-nutritive sucking habits may adversely affect the orofacial complex. This systematic literature review aimed to find scientific evidence on the effect of pacifier sucking on orofacial structures.

**Methods:**

A search on MEDLINE, EMBASE, Cochrane Central Register of Controlled Trials, and Web of Science databases was conducted to find all pertinent articles published from inception until February 2018, based on the Preferred Reporting Items for Systematic Reviews and Meta-Analyses (PRISMA) guidelines. The quality of the studies was evaluated using the risk of bias judgements in non-randomized studies of interventions (ROBINS-I).

**Results:**

Among the 2288 articles found, 17 articles met the selection criteria: seven prospective cohort studies, nine cross-sectional studies, and one randomized clinical trial. Using ROBINS-I, 12 studies were evaluated to have a serious overall risk of bias and five, a moderate one.

These studies claimed a strong association between a pacifier sucking habit and the presence of an anterior open bite and posterior crossbite. Functional/orthodontic pacifiers were shown to cause significantly less open bites than the conventional ones.

**Conclusions:**

High level of evidence of the effect of sucking habits on orofacial structures is missing. The available studies show severe or moderate risk of bias; hence, the findings in the literature need to be very carefully evaluated.

There is moderate evidence that the use of pacifier is associated with anterior open bite and posterior crossbite, thus affecting the harmonious development of orofacial structures.

Functional/orthodontic pacifiers reduce the prevalence of open bite when compared to the conventional ones, but evidence is needed concerning the effects on posterior crossbite. Well-designed randomized controlled trials are needed to further analyze the effects of functional/orthodontic and conventional pacifiers on orofacial structures.

## Background

The use of pacifiers is widespread among babies and children throughout the world. Pacifiers are frequently used to calm crying babies, to increase the well-being of the parents and babies, and to prevent thumb or finger sucking [1, 2]. The use of pacifiers in some developed countries is so culturally established that the prevalence is up to 42.5% in young children by the age of 12 months [3]. Pacifier sucking is a common non-nutritive habit and has received considerable attention over many years [1, 2].

Pacifiers were cited for the first time in medical literature in 1473, being described by German physician Bartholomäus Metlinger in his book “Kinderbüchlein,” retitled on later editions as “Ein Regiment der jungen Kinder” (“A Guide on Young Children”) [4]. Pacifiers consist of a latex or silicone nipple with a firm plastic shield and handle and are available in different forms and sizes. There are many types of pacifiers such as the conventional pacifier NUK® [5], the functional pacifier Dentistar® [6], and the orthodontic pacifier Curaprox Baby® [7]. However, a proper definition for a functional or orthodontic pacifier is missing.

According to non-randomized studies, the use of conventional pacifiers may impair the development of orofacial structures, cause infections, shorten the duration of breast-feeding, and produce dental malocclusions [2, 8, 9].

The effects of the use of pacifiers is duration and frequency dependent [10].

However, the use of pacifiers has been described as having a protective effect against Sudden Infant Death Syndrome [2, 3], but the level of evidence is very low [9], since no RCTs are available that has reliably tested the above mentioned hypothesis [11].

Pacifiers designed to cause less side effects, the so-called orthodontic pacifiers, have been introduced into the market [12–15] or have been used to correct an already existing pacifier-associated open bite and increased overjet [16]. A systematic review comparing conventional and orthodontic nipples could not show significant differences on their effects on the stomatognathic system [9]. However, a recent randomized controlled trial has shown that a thin neck nipple reduces the occurrence of anterior open bite and increased overjet [16].

The purpose of this study was to systematically review the evidence of the scientific literature on the effects of pacifier sucking on orofacial structures, including the evidence of differences between orthodontic and conventional pacifiers.

## Materials and methods

### Search methods

The present systematic review was conducted and reported according to the Preferred Reporting Items for Systematic reviews and Meta Analyses (PRISMA) guidelines for reporting studies to evaluate health care interventions and Cochrane studies method [17–19]. The first phase of this systematic review involved the development of a specific protocol and a research question based on the Population Intervention Control Outcome (PICO) format [20]. The quality of the studies was evaluated using the risk of bias judgements in non-randomized studies of interventions tool (ROBINS-I) [21].

### Data collection and selection of studies

Two reviewers (KS, RK) independently searched the titles and abstracts of the following databases by using a standardized form: MEDLINE (via PubMed), EMBASE, Cochrane Central Register of Controlled Trials, and Web of Science. The keywords used to identify the relevant studies were pacifier, dummy, comforter, tooth, malocclusion, deciduous, tongue, swallowing, openbite, crossbite, myofunctional, muscle, and orofacial, and the different terms were combined using Boolean operators. Full texts of eligible studies were only obtained when both reviewers were in consensus.

Lack of agreement between the reviewers was resolved by discussion with a third reviewer who acted as an arbitrator. The selected studies and relevant articles were checked for cross references. The remaining articles were evaluated by reading the full text independently.

### Selection criteria

The types of studies intended to be included in the search comprised randomized controlled trials (RCTs), cohort studies, intervention studies, case-control studies, and cross-sectional studies. No language restrictions were applied in this search. The minimum number of participants in the relevant subgroups of the study population was 30. There were no limitations on the date of publication or the place where the studies were carried out.

### Type of participants

The participants of the reviewed articles included healthy babies and children who only had a pacifier sucking habit. The studies including subjects with craniofacial anomalies (e.g., cleft lip and palate), systemic diseases, or older infants undergoing or having had previous orthodontic treatment were excluded from this review.

### Type of intervention

The studies which analyzed pacifier sucking effects on orofacial structures were included.

### Type of outcome measures

The effects of pacifier sucking should have measurable documentation relating to any malocclusion and/or other anomalies in orofacial structures.

### Data extraction

The two reviewers extracted data independently using a standardized form. The following factors were recorded when the information in the reviewed articles was available: study design, country where the study was performed, sample size, follow-up, age range, control group characteristics, examined dental and orofacial features with specific reference to anterior open bite, posterior crossbite, overjet, overbite, molar and canine relationship, and arch width. The level of evidence of each study was classified by means of the Oxford Centre of Evidence-Based Medicine [22]. The reviewers resolved any disagreement by means of discussion. In case of insolvable discrepancies, a third reviewer acted as an arbitrator.

### Assessment of the risk of bias

The risk of bias was assessed according to the type of study available. Since only one RCT study was conducted on this topic, the assessment of the risk of bias was performed using the risk of bias in non-randomized studies of intervention (ROBINS-I) tool. It includes risk of bias due to confounding factors (lack of information on type of pacifier, start and finish of the habit, initial malocclusion, presence of digital or object sucking), selection of participants into the study, classification of interventions, deviations from intended intervention, missing data, measurement of outcomes, and selection of the reported results [21].

The reviewers ranked independently each included study and resolved any disagreement by reciprocal consulting.

## Results

### Results of the search

**Fig. 1:**
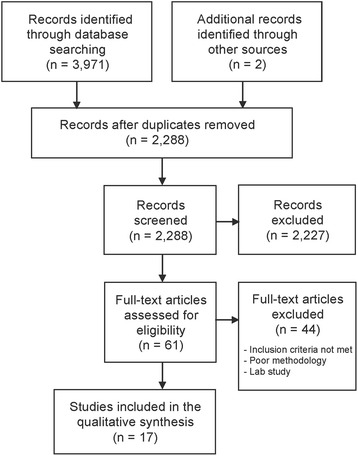
Flowchart of the study selection process

The initial electronic search resulted in 2288 studies after duplicates were removed. After screening the titles and abstracts, 2227 were excluded. From the remaining articles, 61 full-text articles were eligible for assessment. Seventeen articles published up to February 2018 could be included in this systematic literature review (Fig. 1): seven prospective cohort studies, nine cross-sectional studies, and one RCT [3, 8, 10, 12–15, 23–32]. Forty-four articles were excluded; 32 did not meet the inclusion criteria (non-healthy children included, undefined non-nutritive sucking habit, insufficient sample size, incorrect age group), 11 showed poor methodology (no control group, unclear measurement methods, unclear duration habit), and one was a finite element analysis. An overview of the most important data from the included studies is presented in Table 1.

**Table 1:** Summary of characteristics and results of the included studies

Study, year	Study design, country	Sample size	Follow-up	Age range (mean ± SD) in months	Characteristics of the control group	Examined orofacial structures	Results: Pacifier group	Results: Control group
Adair et al., 1995 [12]	Cross-sectional study, USA	218	No	24–59 (44 ± 9)	No sucking habits	AOB, PCB, overjet, molar and canine relationship	AOB: 16.7%**, PCB:15%*, overjet (≥ 4 mm): 20%*, class II primary canine relationship: 17.7%*, distal step molar relationship: 9.6%*	AOB: 3.1%, PCB: 5.1%, overjet (≥ 4 mm): 10.2%, class II primary canine relationship: 9.3%, distal step molar relationship: 3.6%
de Sousa et al., 2014 [23]	Cross-sectional study, Brazil	732	No	36–60	No pacifier sucking habits	AOB, PCB	AOB: 38.6.9%***, PCB: 18.2%***, AOB ≥ 36 months pacifier sucking: 73.5% vs < 36 months pacifier sucking: 19.5%***, PCB ≥ 36 months pacifier sucking: 25.5% vs < 36 months pacifier sucking: 15.5%*	AOB: 5.2%, PCB: 5.7%
Dimberg et al., 2010 [24]	Cross-sectional study, Sweden	457	No	36 ± 3	No sucking habits	AOB, PCB, overjet, class II canine relationship	AOB: 66%***, PCB: 25%***, overjet (> 4 mm): 28%***, class II canine relationship: 34%***	AOB: 1%, PCB: 1%, overjet (> 4 mm): 4%, class II canine relationship: 2%
Duncan et al., 2008 [10]	Prospective cohort study, Great Britain	867	0–61 months of age (questionnaire after 15, 24, and 36 months of age, clinical examination after 31, 43, and 61 months of age)	31, 43, and 61	No sucking habits	AOB, PCB, spaced labial segment	Pacifier sucking at 15 months: presence of AOB at 31, 43, and 61 months**, presence of PCB at 31, 43, and 61 months***, spaced upper labial segment at 61 months***, spaced lower labial segment at 31 months*** Pacifier sucking at 24 months: presence of AOB at 31, 43, and 61 months***, presence of PCB at 31, 43, and 61 months***, alignment of the upper labial segment at 43 and 61 months**, alignment of the lower labial segment at 31 months*** Pacifier sucking at 36 months: presence of AOB at 43 and 61 months***, presence of PCB at 43 and 61 months ***, alignment of the upper labial segment at 61 months***, alignment of the lower labial segment at 43 months***	No data in paper
Facciolli Hebling et al., 2008 [25]	Cross-sectional study, Brazil	728	No	60	No pacifier sucking habits	AOB, PCB	AOB: 81.5%***, PCB: 27.1***	AOB: 11.85%, PCB: 13.73%
Katz and Rosenblatt, 2005 [26]	Prospective cohort study, Brazil	305	1 year	At the beginning average age: 48 ± 5	No sucking habits	AOB	AOB: 72% of the AOB group was pacifier sucking (initial examination) and 59% in the final examination*	AOB: 16% of the AOB group had no sucking habits (initial examination) and 23% in the final examination
Lagana et al., 2013 [27]	Cross-sectional study, Albania	2617	No	84–180	No control group	Molar and canine relationship	Molar and canine relationship: no statistically significant correlation	
Lima et al., 2016 [15]	Prospective cohort study, Brazil	220	Birth up to the age of 29 ± 2 months; T1: birth, T2: questionnaire after 12–24 months, T3: clinical examination at the age of 24–36 months	24–36 (29 ± 2)	No pacifier sucking habits	AOB, PCB, overjet, molar and canine relationship, deep overbite	AOB: 96.3%**, PCB: 88.9%*, overjet (> 2 mm): 67.5%***, distal step of primary molar: 77.8%***, mesial step of primary molar: 6%***, flush: 85.7%***, deep overbite: 77.8%***	AOB: 3.7%, PCB: 11.1%, overjet (> 2 mm): 32.5%, distal step of primary molar: 22.2%, mesial step of primary molar: 64%, flush: 22.8%, deep overbite: 22.2%
Melsen et al., 1979 [28]	Cross-sectional study, Denmark	723	No	120–132	No sucking habits	AOB, PCB, overjet, molar and canine relationship, swallowing (normal swallow (NSW), tongue-thrust swallow (TTS), teeth-apart swallow (TAS), deep overbite	AOB: 8.5%, NSW: 0.6%, TTS: 34%, TAS: 12.4%, PCB: 14.1%***, NSW: 12.4%, TTS: 14.9%***, TAS: 17.1%*, increased overjet: 23.3%, NSW: 13.9%, TTS: 51.1%, TAS: 26%, bilateral distal occlusion: 41.4%***, NSW: 24.5%, TTS: 74.5%, TAS: 56.3%, mesial occlusion: 1.9%**, NSW: 2.2%**, TTS: 1.1%, TAS: 1.9%, deep overbite: 32%, NSW: 34.1%*, TTS: 22.3%, TAS: 33.5%	AOB: 5.2%, NSW: 1.4%, TTS: 25%, TAS: 9.5%, PCB: 3.1%, NSW: 2.8%, TTS: 0%, TAS: 4.7%, increased overjet: 15.9%, NSW: 7.4%, TTS: 50%, TAS: 38.5%, bilateral distal occlusion: 24%, NSW: 14.9%, TTS: 62.5%, TAS: 38.1%, mesial occlusion: 0%, NSW: 0%, TTS: 0%, TAS: 0%, Deep overbite: 25%, NSW: 22.4%, TTS: 11.7%, TAS: 38.1%
Moimaz et al., 2014 [3]	Prospective cohort study, Brazil	80	Birth up to 30 months of age (questionnaire after 12, 18, and 30 months of age, clinical examination at the 30 months of age	30	No control group	AOB, PCB, overjet, overbite (> 3 mm)	AOB and overjet (> 3 mm): pacifier sucking stop at 12***, 18, and 30***months, PCB: pacifier sucking stop at 12, 18, and 30 months, overbite (> 3 mm): pacifier sucking stop at 12, 18, and 30** months	
Scavone et al., 2007 [29]	Cross-sectional study, Brazil	366	No	36–72	No pacifier- and finger sucking habits	PCB	PCB: 20.4%**, pacifier sucking until 2 years of age: 17.2%*, pacifier sucking until 4 years of age: 16.9%*, pacifier sucking until 6 years of age: 27.3%**	PCB: 5.2%
Schlomer, 1984 [30]	Cross-sectional study, Germany	582	No	36–72	No sucking habits	AOB, PCB, overjet (no significance data in the study) (no significance data in the study at all)	AOB: 22.8%, PCB: 12.8%, overjet (> 3 mm): 13.2%, pacifier sucking (NUK) stop until 3 years: 3–4 years of age: AOB: 15.2%, PCB: 23.9%, overjet (> 3 mm):10.9%, 5 years of age: AOB: 9.6%, PCB: 11.5%, overjet (> 3 mm): 1.9%, 6 years pf age: AOB: 2.7%, PCB: 10.8%, overjet (> 3 mm): 5.4% (no statistical significance for all mentioned data)	AOB: 2.1%, PCB 0.7%, Overjet (> 3 mm): 12.2%
Tibolla et al., 2012 [31]	Cross-sectional study, Brazil	237	No	36–168: Deciduous: 36–60, Mixed: 72–120, Permanent: 132–168	No pacifier sucking habit	AOB	AOB: 36.5%***, deciduous dentition: 65%***, mixed dentition: 37.3%**, permanent dentition: 23.4%*, > 2 years pacifier sucking vs ≤ 2 years: in deciduous dentition: 83.3% vs. 11.5%***, mixed dentition: 47.6% vs. 11.7***, permanent dentition: 25.9 vs. 9.4%, no or/and only to sleep vs. all the time in deciduous dentition: 17.2% vs. 88.9%***, in mixed dentition: 15.7% vs. 44.4%**, in permanent dentition: 10.7% vs. 25%	AOB: 7.2%, deciduous: 0%, mixed: 11.7%, permanent: 3%
Wagner and Heinrich-Weltzien [16]	Randomized clinical trial, Germany	86	3, 6, 9, and 12 months	16–24 months (20.3)	Weaned of pacifier with a pacifier-associated open bite or overjet ≥ 2 mm	AOB, overjet	Overjet 2.7 ± 0.5 mm, AOB − 1.2 ± 0.3 mm	Overjet 2.4 ± 0.5 mm, AOB − 0.8 ± 0.8 mm
Warren and Bishara, 2002 [32]	Prospective cohort study, USA	372	Birth until 5 years (questionnaire after 3, 6, 9, 12, 20, and 24 months of age, after that yearly, clinical examination: 54–60 months age)	54–60	No control group	AOB, PCB, overjet, molar and canine relationship, vertical overbite, arch width	AOB: 24–36 months pacifier sucking: lower prevalence than < 12 months**, PCB: 24–36 months had a higher prevalence of PCB than ≤24 months**, overjet (≥4 mm): 24–36 months pacifier sucking: lower prevalence than < 12 months, class II canine relationship: 24–36 months pacifier sucking: slightly higher prevalence than < 12 months, arch width and depths measurements: only small differences between < 12 months and 12–36 months pacifier sucking, vertical overbite (mm): not statistically significant between pacifier sucking less than 12 months, 12 to 24 months, and 24 to 36 months.	
Zimmer et al., 2011 [13]	Prospective cohort study, Germany	121	0–26 months	16 ± 4	No pacifier sucking habit	AOB, overjet	AOB: NUK-group: 38%***, Dentistar group: 5%, overjet (mm): NUK-group: 1.7 ± 1.4, Dentistar group: 1.3 ± 1.0	AOB: 0%, overjet: 1 ± 1 mm
Zimmer et al., 2016 [14]	Prospective cohort study, Germany	121	12 months	20–36	No pacifier sucking habit	AOB, overjet, molar and canine relationship, deep overbite	AOB: NUK-group: 50%, Dentistar group: 6.7%, increased overjet: NUK-group: 19%, Dentistar group: 31.1%**, class II primary canine and molar relationship: NUK-group: 11.1%, Dentistar group: 4.8%, deep overbite: NUK-group: 2.4%, Dentistar group: 6.7%,	AOB: 0%, increased overjet: 5.9%, class II primary canine and molar relationship: 2.9%, deep overbite: 8.9%

*AOB* anterior open bite, *PCB* posterior crossbite, *NSW* normal swallow, *TTS* tongue-thrust swallow, *TAS* teeth-apart swallow

*p < 0.05; **p < 0.01; ***p < 0.001

### Risk of bias of included studies

The risk of bias judgements in ROBINS-I including pre-, at-, and post-intervention domains are depicted in Table 2. Eleven non-randomized studies were evaluated to have an overall serious risk of bias, and four non-randomized studies and one RTC study had a moderate overall risk of bias.

**Table 2:** ROBINS-I (risk of bias judgements in non-randomized studies of interventions)

	Confounding	Selection of participants	Classification of interventions	Deviations from intended interventions	Missing data	Measurement of outcomes	Selection of reported results	Overall
Adair et al., 1995 [12]	Serious	Serious	Moderate	Moderate	Moderate	Low	Low	Serious
de Sousa et al., 2014 [23]	Serious	Serious	Moderate	Moderate	Moderate	Low	Low	Serious
Dimberg et al., 2010 [24]	Serious	Low	Moderate	Moderate	Moderate	Moderate	Moderate	Serious
Duncan et al., 2008 [9]	Serious	Moderate	Moderate	Moderate	Moderate	Moderate	Moderate	Serious
Facciolli Hebling et al., 2008 [25]	Serious	Serious	Moderate	Serious	Moderate	Moderate	Moderate	Serious
Katz and Rosenblatt, 2005 [26]	Moderate	Serious	Moderate	Moderate	Low	Low	Moderate	Serious
Lagana et al., 2013 [27]	Serious	Serious	Moderate	Serious	Moderate	Moderate	Moderate	Serious
Lima et al., 2016 [15]	Moderate	Moderate	Low	Moderate	Moderate	Low	Moderate	Moderate
Melsen et al., 1979 [28]	Serious	Serious	Moderate	Serious	Moderate	Moderate	Moderate	Serious
Moimaz et al., 2014 [3]	Moderate	Moderate	Low	Low	Low	Low	Low	Moderate
Scavone et al., 2007 [29]	Moderate	Serious	Moderate	Moderate	Moderate	Moderate	Moderate	Serious
Schlomer, 1984 [30]	Serious	Serious	Serious	Serious	Serious	Serious	Moderate	Serious
Tibolla et al., 2012 [31]	Serious	Serious	Serious	Serious	Serious	Moderate	Moderate	Serious
Wagner and Heinrich-Weltzien, 2016 [16]	Low	Low	Low	Low	Moderate	Low	Moderate	Moderate
Warren and Bishara, 2002 [32]	Low	Low	Low	Low	Moderate	Low	Low	Moderate
Zimmer et al., 2011 [13]	Moderate	Moderate	Low	Moderate	Moderate	Low	Low	Moderate
Zimmer et al., 2016 [14]	Serious	Moderate	Moderate	Moderate	Moderate	Low	Low	Serious

*Low* comparable to a well-performed randomized trial, *Moderate* sound for a non-randomized study, but not comparable to a rigorous randomized trial, *Serious* presence of important problems, *Critical* too problematic to provide any useful evidence on the effects of intervention, *Overall risk of bias* equal to the most severe level of bias found in any domain

### Effects of pacifier on anterior open bite (AOB)

The prevalence of AOB in children using pacifier varies between 8.5 [28] and 96.3% [15]. It is worth mentioning that not all studies analyzed the same age groups nor the same type of pacifiers (Table 1).

Fifteen out of the reviewed 17 articles showed a strong association between AOB and the use of a pacifier when compared with the children not using the pacifier [3, 10, 12–16, 23–26, 28, 30–32]. Duration and frequency of pacifier sucking played an important role [10, 23, 31, 32]. The use of pacifier for more than 36 months was associated with AOB [10, 23]. Two studies showed that children who used a pacifier for more than 2 years were more likely to develop an AOB than children who used it for less than 2 years [10, 31]. One study showed that discontinuing the use of pacifier at 1 year of age may still result in an anterior open bite; however, this study had a serious risk of bias (Table 2) [3]. Tibolla et al. found that the presence of anterior open bite was statistically significantly higher in children using a pacifier during the day and night when compared to use at nighttime only [31]. However, Dimberg et al. could not support the above mentioned finding [24].

### Effects of pacifier on posterior crossbite (PCB)

The PCB prevalence in children using pacifier varies between 12.8 [30] and 88.9% [15]. The use of pacifiers can lead to posterior crossbite according to nine of the reviewed articles [10, 12, 15, 23–25, 28–30]. However, Moimaz et al. could not find a statistically significant difference concerning posterior crossbite between the patients that used and those who did not use a pacifier at 12, 18, and 30 months except when the posterior crossbite was associated with finger sucking [3]. According to Scavone et al., the children who discontinued pacifier sucking by 2 years of age presented a lower prevalence of posterior crossbite (17.2%) than the ones that continued the pacifier sucking until 4 to 6 years of age (27.3%) [29]. De Sousa et al. showed that 25.5% of the children who used a pacifier for more than 36 months developed posterior crossbite compared to only 15.5% of the children who stopped pacifier sucking before 36 months of age [23]. However, both latter two studies presented a severe risk of bias.

According to Dimberg et al., 17% of the children who used a pacifier during the day had a posterior crossbite, compared to 23% of the children who used it at night and 31% of the children who used the pacifier during both day and night [24].

### Effects of pacifier on overjet

Studies with severe and moderate risk of bias have shown that the prevalence of overjet is increased in children using a pacifier when compared with children who do not use a pacifier. Dimberg et al. found that an overjet of more than 4 mm was present in 28% of the pacifier users, compared with only 4% of children without any sucking habits [24]. Adair et al. reported that pacifier users had a higher average overjet of 4 mm (20%) than the group without a pacifier sucking habit (10.2%) [12]. A higher prevalence of overjet was associated with a pacifier sucking habit at 12, 18, and 30 months after birth [3]. Lima et al. found that the prevalence of overjet (> 2 mm) was higher in children with a pacifier sucking habit (67.5%) than in children who did not use a pacifier (35.5%) [15]. Other studies also found an association between pacifier sucking and increased overjet [13, 28, 32].

### Effects of pacifier on molar and canine relationships

A distal molar and canine relationship was found in patients using a pacifier [12, 15, 24, 28]. Adair et al. and Dimberg et al. showed that distal molar and canine relationship had a statistically significant higher occurrence in patients using a pacifier than in children without using one [12, 15], whereas two studies have not found any strong association [14, 32].

Sagittal canine relationship showed no statistically significant differences in patients with a pacifier sucking habit when grouped according to habit duration [32].

### Effect of pacifier on the dental arch

Warren and Bishara examined the effect of the duration of pacifier use on different dental arch measurements and found a statistically significant increased mandibular canine arch width and a statistically significant decrease in palatal depths [32].

### Effect of pacifier on swallowing

The only study dealing with swallowing pattern was the one of Melsen et al. that presented a severe risk of bias. They found that the prevalence of abnormal swallowing patterns was higher in children with a pacifier habit. The majority of subjects using a pacifier had a normal swallowing (56.2%), whilst simple tongue-thrust occurred in 16.3% and complex tongue thrust in 27.5%. In the non-pacifier group, 69.8% of the children presented with a normal swallowing pattern and simple tongue thrust occurred only in 8.3% and complex tongue thrust in 21.9% [28].

### Effect of different types of pacifier on orofacial structures

Among the five studies that assessed the effect of different types of pacifiers on the orofacial structures, three were classified as having a moderate overall risk of bias [13, 15, 16] and two a serious one [12, 14]. Functional orthodontic pacifiers seem to cause less dental malocclusion than conventional ones (Table 3) [12–16].

**Table 3:** Effects of different types of pacifiers on orofacial structures

Study	Age of examination in months	Pacifier type 1	Pacifier type 2	Examined orofacial structures	Results (%)		Statistical significance (*p* value)
					Pacifier type 1	Pacifier type 2	
Adair et al., 1995 [12]	24–59 (43.9)	Functional exerciser	Conventional pacifier	AOB	13.4	23.7	0.19
				PCB	15.9	13.2	0.79
				Overjet (≥4 mm)	23.2	13.2	0.23
				Class II primary canine relationship	26.8	5.3	0.01
				Class II primary molar relationship	15.9	2.6	0.04
Lima et al., 2016 [15]	24–36 (29.0 (±2.0 SD))	Orthodontic pacifier	Conventional pacifier	AOB	44.3	55.7	0.03
				PCB	37.5	62.5	0.72
				Overjet (> 2 mm)	42.9	57.1	0.11
				Class II primary molar relationship	28.6	71.4	0.78
				Flush of primary molar relationship	41.7	58.3	0.78
				Deep overbite	64.3	35.7	0.23
				Diastema	55.6	44.4	0.32
				Crowding	66.7	33.3	> 0.999
Wagner and Heinrich-Weltzien, 2016 [16]	16–24 months (20.3)	Thin neck pacifier (TNP)	Conventional or physiological	AOB Overjet (≥2 mm)	−1.2 2.7	−2.2 3.2	< 0.001 < 0.001
Zimmer et al., 2011 [13]	15.9 (± 3.9 SD)	Dentistar	NUK	AOB	5	38	< 0.001
				Overjet (mm), mean ± SD	1.3 ± 1.0	1.7 ± 1.4	> 0.05
Zimmer et al., 2016 [14]	20–36 months	Dentistar	NUK	AOB	6.7	50	0.00
				Increased overjet	31.1	19.0	0.23
				Class II primary canine and molar relationship	4.8	11.1	0.29
				Deep overbite	6.7	2.4	0.47

*AOB* anterior open bite, *PCB* posterior crossbite

Wagner et al. found that the use of a thin neck pacifier decreases the occurrence of open bite and increased overjet [16]. The prevalence of anterior open bite was higher in children who used a NUK® pacifier compared to the Dentistar® pacifier, the latter having a thin neck [13, 14]. The same authors found an increased overjet in 31.1% of the children who used a Dentistar® pacifier and only in 19% of probands using the NUK® pacifier [14]. Lima et al. showed that the prevalence of anterior open bite, overjet, posterior crossbite, and distal step of primary molars were larger in children who used conventional pacifiers compared to orthodontic pacifiers [15]. Children using a conventional pacifier presented an increased prevalence of anterior open bite when compared to children using a functional exerciser. However, no statistically significant differences were found in the prevalence of posterior crossbite between these two groups of pacifiers. The prevalence of an overjet of 4 mm or more and the occurrence of distal primary molar and canine relationships was higher in children using a functional exerciser when compared to the group of children using a conventional pacifier [12].

### Self-correction of malocclusion

In a cross-sectional follow-up study, Katz and Rosenblatt found that 23 out of 30 children who discontinued the non-nutritive sucking showed a self-correction of the open bite. However, self-correction of an open bite was observed also in children (6 out of 17) without non-nutritive sucking habits [26].

## Discussion

This systematic review revealed a lack of high-quality evidence in publications dealing with the effects of pacifier sucking habits on orofacial structures.

In the hierarchy of evidence, the randomized controlled trials are on the top, cross-sectional studies come in the middle, and retrospective studies are at the bottom [11]. A single randomized clinical trial was found in the literature showing an improvement of the overjet and overbite in children presenting a pacifier-associated open bite and increased overjet when using a thin neck pacifier, when comparing with children still using the usual pacifier or discontinuing the habit [16]. In our review we needed to include non-randomized cohort studies and cross-sectional studies due to the lack of sufficient number of randomized controlled trials examining the association between pacifier sucking and its effects on the orofacial structures. We considered as cohort studies also those without a control group, as defined by Dekkers et al. [33]. The risk of bias of non-randomized cohort and cross-sectional studies was assessed to be from moderate to serious, and the conclusion of those studies has to be considered with substantial caution when analyzing the findings of the studies included in this systematic review of the literature (see Table 2).

The ROBINS-I assessment tool is based on the Cochrane RoB tool for randomized trials and was used due to the fact that we found in our search only one study that was a true randomized trial. Other studies were defined as randomized according to the authors, but a thorough review revealed in reality severe risks of bias. The ROBINS-I assessment tool uses the domain-based assessment and has a comprehensive manual in which users can interpret the results in a similar way, thus reducing the risk of subjective evaluation.

Our assessment is in accordance with the findings of Dogramaci and Rossi-Fedele [8], despite the fact that we focused only on the effect of pacifiers and not on non-nutritive sucking habits in general. They concluded that the use of pacifiers in the deciduous dentition is cause of malocclusions [8]. Our review, although with moderate and serious overall risk of bias, shows that the use of pacifiers seems to be associated with anterior open bite [12–15, 23–26, 30, 31] and posterior crossbite [10, 12, 15, 23–25, 28–30].

We focused specifically on the influence of pacifiers on orofacial structures, since the so-called functional/orthodontic pacifiers seem to be a promising tool to limit their negative influence on the occlusion [7, 13, 14, 16]. However, the lack of sufficient number and quality of randomized clinical trials on the effect of pacifiers on the occlusion weakens the evidence. On the other hand, it has to be taken into consideration that well-designed randomized clinical trials might raise ethical concerns and interactions with other factors may make the control of concomitant variables difficult [34]. The only systematic literature review available on the difference between conventional and orthodontic nipples was not able to draw any conclusion due to the low level of evidence of the available studies [9]. However, Wagner and Heinrich-Weltzien have recently shown in a randomized clinical trial that a thin neck nipple reduces the occurrence of open bite and increased overjet [16]. The effect of the nipple on the transverse dimension was assessed by Adair and Lima et al. who found that the functional/orthodontic pacifiers under investigation do not induce less posterior crossbites than the conventional ones [12, 15].

The duration and frequency of pacifier sucking was not examined in all the studies, although playing a role in the developing of malocclusions [10, 23, 31, 32]. The data regarding the duration often relies on the information provided by parents or guardians, and therefore, the uniformity of data collection could be affected. Tools able to quantify the duration of pacifier use should be developed in order to properly control the time factor on the generation of malocclusion traits.

Different studies disagreed on the correlation between pacifier use and increased overjet [3, 12, 13, 15, 24, 28, 32], and the majority of them showed a serious overall risk of bias.

## Conclusions

A high level of evidence on the effects of pacifier habits on orofacial structures is non-existent.

However, there is moderate evidence that the use of pacifier is associated with anterior open bite and posterior crossbite, thus affecting the harmonious development of orofacial structures.

Taking into consideration the present evidence, it seems that pacifiers with thin neck induce less open bite than the conventional ones. Functional/orthodontic pacifiers investigated in the literature seem not to reduce the occurrence of posterior crossbite. New nipple shape and texture are needed to counteract the narrowing of the palate.

Randomized controlled trials are strongly needed to further analyze the effect of conventional and functional/orthodontic pacifiers on orofacial structures.

## Abbreviations

AOB: Anterior open bite
PCB: Posterior crossbite
PICO: Population Intervention Control Outcome
PRISMA: Preferred Reporting Items for Systematic reviews and Meta Analyses
RCT: Randomized controlled trials
ROBIN-I: Risk of bias in non-randomized studies of interventions
